# MRL and SuperFine+MRL: new supertree methods

**DOI:** 10.1186/1748-7188-7-3

**Published:** 2012-01-26

**Authors:** Nam Nguyen, Siavash Mirarab, Tandy Warnow

**Affiliations:** 1 Department of Computer Science, University of Texas at Austin, Austin, Texas, USA

**Keywords:** MRP, MRL, supertrees, phylogenetics

## Abstract

**Background:**

Supertree methods combine trees on subsets of the full taxon set together to produce a tree on the entire set of taxa. Of the many supertree methods, the most popular is MRP (Matrix Representation with Parsimony), a method that operates by first encoding the input set of source trees by a large matrix (the "MRP matrix") over {0,1, ?}, and then running maximum parsimony heuristics on the MRP matrix. Experimental studies evaluating MRP in comparison to other supertree methods have established that for large datasets, MRP generally produces trees of equal or greater accuracy than other methods, and can run on larger datasets. A recent development in supertree methods is SuperFine+MRP, a method that combines MRP with a divide-and-conquer approach, and produces more accurate trees in less time than MRP. In this paper we consider a new approach for supertree estimation, called MRL (Matrix Representation with Likelihood). MRL begins with the same MRP matrix, but then analyzes the MRP matrix using heuristics (such as RAxML) for 2-state Maximum Likelihood.

**Results:**

We compared MRP and SuperFine+MRP with MRL and SuperFine+MRL on simulated and biological datasets. We examined the MRP and MRL scores of each method on a wide range of datasets, as well as the resulting topological accuracy of the trees. Our experimental results show that MRL, coupled with a very good ML heuristic such as RAxML, produced more accurate trees than MRP, and MRL scores were more strongly correlated with topological accuracy than MRP scores.

**Conclusions:**

SuperFine+MRP, when based upon a good MP heuristic, such as TNT, produces among the best scores for both MRP and MRL, and is generally faster and more topologically accurate than other supertree methods we tested.

## Background

Because estimation of large trees is computationally challenging [1-3] and topological error tends to increase with the number of taxa [4-7], supertree methods (which estimate trees on full sets of taxa from sets of smaller trees) may be key to accurate estimations of the Tree of Life. Many supertree methods have been proposed: see [8] for an overview of early methods, and also [9-17]. Some of these (e.g., the Robinson-Foulds supertree approach in [9]) operate only on rooted source trees, while others (e.g., the Maximum Likelihood Supertree Method in [15]) are only theoretical (i.e., have not yet been implemented). Of the various methods that are implemented, MRP (Matrix Representation with Parsimony) [18,19] is by far the most frequently used. Furthermore, studies have shown that of these methods, only MRP produces highly accurate supertrees on datasets of unrooted source trees with large numbers of taxa [17,20].

MRP operates in two steps. Given a set T of source trees with set *S *of taxa, the first step produces a large matrix, called the MRP matrix, with one row for every taxon in *S *and one column for every edge of every tree in T. For a given edge *e *in a given source tree *t*, the column in the MRP matrix has entries over {0,1, ?}, with 0 given for the taxa that are on one side of the edge *e*, 1 for the taxa on the other side, and ? for all the remaining taxa (i.e., the ones that do not appear in the tree *t*). This way of encoding each source tree is called the "Baum-Ragan" coding; however, when the source trees are rooted, other techniques (e.g., the Purvis coding) can be used (see a comparison between these coding methods in [20]). The second step then uses the maximum parsimony criterion to produce a tree on the MRP matrix. The MRP approach thus depends on whether substitutions from 0 to 1 have the same cost as substitutions from 1 to 0, and hence also depends on how the states 0 and 1 are assigned to the leaves in each source tree, for each given edge. In particular, the MRP matrix definition depends upon whether the trees in T are rooted or unrooted. The simplest version of MRP (and the one we study in this paper) treats all input trees as unrooted and uses the standard maximum parsimony criterion in which all substitutions have equal cost (this is called "reversible Fitch parsimony"). For this very simple version of MRP (i.e., Baum-Ragan encoding, followed by reversible Fitch parsimony), the choice of state (i.e., 0 or 1) for each side of each edge has no impact on the output, and so can be made arbitrarily. Methods for MRP are based upon techniques for the NP-hard maximum parsimony problem [21]. The most popular MRP heuristics therefore use good heuristics for maximum parsimony (MP), such as PAUP* [22] and TNT [23].

Recently, Swenson et al. [24] introduced a new supertree method, SuperFine+MRP, that operates in two steps: in the first step, an incompletely resolved tree called the "strict consensus merger" (SCM) tree is computed, and in the second step MRP heuristics are used to refine each high degree node (polytomy) in the SCM tree. Their study showed that SuperFine+MRP produced topologically more accurate trees than MRP (both methods based upon the same MP heuristics in PAUP* [22]) and also ran in much less time. However, in some cases (in particular on very large supertree datasets), the SCM tree contained very large polytomies, so that refining the large polytomies could require a substantial time effort, thus reducing the running time advantage of SuperFine+MRP over MRP. Speeding up the analysis through a parallelization of SuperFine's refinement step is also hampered by the fact that refinement of very large polytomies using MRP is the most computationally intensive part of the SuperFine analysis [25].

Our objective was therefore to find an alternative to MRP for the refinement step within SuperFine. In this paper we examine supertrees estimated by analyzing the MRP matrix using RAxML's [26] fast heuristics for maximum likelihood under the symmetric 2-state model (so that the change from 0 to 1 is as likely as the change from 1 to 0) with CAT distribution of rates across sites, and we refer to this as the S2+CAT model. We call the optimization problem in this approach to supertree estimation "matrix representation with likelihood", or MRL. Thus, MRL is the counterpart to MRP, and uses S2+CAT maximum likelihood instead of maximum parsimony as a criterion for estimating a supertree from the MRP matrix.

We report on a simulation study we performed to compare MRP to MRL (using fast heuristics for both) as supertree methods, and also to refine the SCM tree computed by SuperFine. Our study shows that using RAxML for MRL produces topologically more accurate trees than the MRP heuristics (PAUP* and TNT [23]) we studied, and that MRL scores (under GTR+gamma, see discussion below) correlate very well with tree accuracy (and in general better than MRP scores). These results suggest that MRL may be a useful optimality criterion for supertree estimation. Second, we show that SuperFine can be used to obtain better scores for MRP and good scores for MRL, and faster than the standard heuristics for these problems.

## Methods and Materials

### Datasets

#### Simulated datasets

We used 500- and 1000-taxon datasets used in previous studies [17,28]. These supertree datasets consist of profiles of source trees, with each source tree computed by running RAxML [29] on DNA sequence alignments produced in simulation. These simulated datasets have realistic patterns of missing data, reflecting both biological processes and taxon sampling strategies used by systematists in phylogenetic studies. Two types of source trees were generated on the model trees: "clade-based source trees (each tree being a dense sample within a specific clade of the model tree), and "scaffold" source trees (a random sampling of a proportion of the taxa throughout the model tree). The proportion of taxa from the model tree that is sampled in the scaffold tree is called the "scaffold density". Supertrees are generally more accurate when estimated from dense rather than sparse scaffold trees. These simulated datasets have scaffold trees with four densities, 20%, 50%, 75% and 100%. Each supertree dataset consists of a number of clade-based source trees and one scaffold-based source tree, but the number of clade-based source trees depended upon the number of taxa (15 for the 500-taxon datasets and 25 for the 1000-taxon datasets). For each scaffold density, there are 30 replicates with 500 taxa and 10 replicates with 1000 taxa. However, for scaffolds with low densities, a few of the datasets did not have sufficient taxonomic overlap to perform an accurate supertree analysis and were excluded from the results. In total, 4 of the 40 1000-taxon datasets (3 from the 20% scaffold density and 1 from the 50% scaffold density) and 6 of the 120 500-taxon datasets (all from the 20% scaffold density) were excluded from analysis.

#### Biological datasets

We examined the performance of the supertree methods on six biological datasets shown in Table 1. The number of source trees and taxa in each dataset varied from a dataset with a small number of taxa but many source trees (115 taxa and 726 source trees) to a dataset with a large number of taxa but fewer source trees (2,228 taxa and 39 source trees). The details for the generation of the seabirds, placental mammals, marsupial, and THPL source trees can be found in the references mentioned in Table 1. The comprehensive papilionoid legumes (CPL) dataset was originally studied in [30] as a combined analyses of 39 markers and consisted of 2228 taxa and 33,168 sites in the alignment; for this dataset, we used source trees that were estimated by Swenson et al. [17].

**Table 1:** Statistics for biological datasets

Dataset	Number Taxa	Number Source trees	Scaffold density	Resolution of SCM tree	Reference
Placental	116	726	1.00	0.01	[35]
Seabirds	121	7	0.74	0.57	[36]
Marsupials	267	158	1.00	0.10	[37]
THPL	558	19	0.25	0.57	[38]
CPL	2,228	39	0.74	0.52	[30]

We show the number of source trees, total taxa, resolution of the strict consensus merger tree, and the source of the original data for each of the biological datasets. The scaffold density is the proportion of the total taxa present in the largest source tree.

### Supertree methods

Since earlier studies [17,28] established that the simplest version of MRP (unrooted source trees, all substitutions have equal cost) outperformed other base supertree methods, we used MRP as a benchmark.

#### MRP

For the MP heuristic used within MRP analyses, we ran PAUP* using the parsimony ratchet implementation, identically as in [17,28]. We also ran TNT [23], using a combination of sectorial search, tree drifting, and fusing (i.e., techniques within TNT that are effective for large-scale parsimony analysis). We refer to these two ways to run MRP as MRP(PAUP*) and MRP(TNT). At the end of each MRP analysis, we had a collection of equally good MRP solutions, from which we produced a greedy consensus tree (also known as an extended majority consensus). Scripts used to generate the PAUP* and TNT runs are available upon request.

#### MRL

For the ML heuristic used within the MRL analyses, we used RAxML [29]. RAxML is potentially the most accurate ML heuristic for large datasets, and when used with its BINCAT model it can work directly with the MRP matrices. We refer to this way of running MRL by MRL(RAxML). Note that any ML package that supports the symmetric 2-state model can be used instead of RAxML for the MRL analysis.

#### SuperFine

We briefly describe SuperFine [24]. SuperFine uses a two-step technique, where the first step produces a typically unresolved tree called the "Strict Consensus Merger" (SCM) tree, and the second step then refines this tree. The SCM tree is obtained by merging two trees at a time until all the trees are combined. Each of these pairwise mergers contracts edges on which the two trees either disagree or which have "collisions". At the end of the process, the final tree contains all the taxa, but may be only partially resolved. The order in which the trees are merged can impact the accuracy (and resolution) of the final tree, as observed in [31]; therefore, we use the same rule for determining the ordering on pairwise mergers as used in [24], which considers the size of the overlap in taxon sets when picking the pair of trees to merge.

The second step of SuperFine resolves the SCM tree, one node at a time. The resolution of a single high degree node (described below) depends only on the partition of the taxa into subsets, as defined by the node, and the topologies of the source trees; therefore, these resolutions are independent of each other and so the order does not matter.

To resolve a single high degree node *v *(i.e., polytomy) in the SCM tree, SuperFine first labels the neighbors of *v *by 1...*d*, where the SCM tree has *d *subtrees off of *v *(i.e., *d *= *deg*(*v*)). Next every leaf in each of *d *subtrees is relabeled by the label (from 1...*d*) assigned to the root of its subtree. At this point, SuperFine creates a new set of source trees, by modifying each of the input source trees so that each contains at most *d *leaves, as follows: If *x *is an internal node in a source tree that is adjacent to two leaves, each of which has the same label *l*, then we remove its neighboring leaves and relabel *x *by *l*. In [24], it was proven that this modification produces source trees that have at most one leaf with each label. SuperFine then applies its base supertree method (such as MRP) to compute a supertree on the modified source trees, each of which has at most *d *leaves. Since *d *may be much smaller than the number of taxa, this supertree estimation can be very fast. Finally, the resultant supertree produced for this polytomy is used to define the refinement at that node. Note, therefore, that the refinement at that node depends only on the base supertree method and the modified source trees, and that the modified source trees are defined by the source trees and the partition of the taxa defined by the node. Because the source trees and the partition of the taxa defined by a node *v *does not depend upon whether other polytomies are processed before or after *v*, the order in which the polytomies are processed has no impact on the output.

We use the terminology "SuperFine+MRP" to refer to SuperFine used with MRP to perform the refinement step, but SuperFine can be used with other base supertree methods. Thus, we use the terminology "SuperFine+MRL" to refer to SuperFine used with MRL to perform the refinement step. We ran SuperFine+MRP based upon PAUP* (using the same parsimony ratchet implementation as MRP(PAUP*)) and TNT (using the same combination of sectorial search, tree drifting, and fusing as MRP(TNT)), and refer to these two different versions by SuperFine+MRP(PAUP*) and SuperFine+MRP(TNT). We note that this way of running SuperFine+MRP(PAUP*) is identical to that reported in [24]. We ran SuperFine+MRL based upon RAxML (using the same RAxML commands as MRL(RAxML)), and refer to this as SuperFine+MRL(RAxML).

The software used in this study is available in open-source form by request from the authors; the datasets are available online at http://www.cs.utexas.edu/users/phylo/datasets/supertrees.html.

### Measurements

We compared the trees produced by the supertree methods (MRP, MRL, SuperFine+MRP, and SuperFine+MRL) to the true supertree (known because the data are simulated). We report two error rates: the missing branch rate (i.e., the percent of the internal edges in the true supertree missing in the estimated supertree, and also known as the false negative (FN) rate) and the false positive rate (i.e., the percentage of the internal edges in the estimated supertree that do not appear in the true supertree). For those estimated supertrees that are fully resolved, these two error rates will be equal. However, the false positive error will always be at most that of the false negative error rate, since the true supertree is always binary.

We computed the MRP scores of the estimated supertrees (i.e., the MP scores of the trees with respect to the MRP matrix). We report ML scores under S2+Γ (the symmetric 2-state model with gamma distribution of rates across sites) instead of under the S2+CAT model. This is motivated by the observation that RAxML's search under S2+Γ is computationally more intensive than its search under S2+CAT, and that both searches return trees of comparable topological accuracy. However, the preferred models for phylogeny estimation have used gamma-distributed rates instead of CAT-distributed rates. For these two reasons, we report ML scores under gamma-distributed rates for trees found using the S2+CAT model, and we call these the "MRL scores". Finally, since MRP methods return a set of most parsimonious trees, we report the false positive rates, false negative rates, and MRL scores with respect to the greedy consensus of the set of trees. For the MRP scores, we report the best MP score found during the heuristic search.

For the biological datasets, since the true supertrees are not known, we computed the Sum-FN [17] distance to the source trees, which is the percent of the internal edges in the source trees missing in the estimated supertree. Note that Sum-FN is identical to Sum-RF (the sum of the RF distances) when the source trees and the estimated supertree are binary. We also computed the MRP and MRL scores of the estimated supertrees.

Finally, we also report average running times for each method on each model condition as well as on the biological datasets.

### Correlation Analyses

For each simulated dataset, we examined how well each of the scores (MRP score, MRL score, and Sum-FN) correlate with missing branch rates. Since we only have six estimated supertrees per dataset, we generated more trees for each dataset to run our correlation analyses, using *p*-edge-contract-and-refine (*p*-ECR) [32] moves as follows. A *p*-ECR move operates by randomly contracting *p *edges in a tree and then randomly refining the resultant unresolved tree. For each dataset in each model condition, the six estimated supertrees were used to generate a set of p-ECR neighboring trees, with *p *drawn between 0 and 25% of the internal edges. This was repeated 100 times per supertree, resulting in a total of 600 trees. We then compute the MRL, MRP, Sum-FN, and missing branch rates for each of the 606 trees (600 *p*-ECR trees plus 6 supertrees). In other words, the results we report are for 114 different 500-taxon supertree datasets (24 from the 20% scaffold density and 30 from the remaining three model conditions) and 36 different 1000-taxon datasets (7 from the 20% scaffold density, 9 from the 50% scaffold density, and 10 from the remaining two model conditions). We compute the Spearman's rank correlation between the MRL, MRP, and Sum-FN scores and the missing branch rates. We then averaged the Spearman's rank correlation coefficient across replicates and report the average Spearman's rank correlation as a function of the model condition.

## Results

### Simulated Datasets

We begin our discussion of results by examining the results for the simulated datasets. We focus primarily on the average missing branch rates for each model condition (as defined by the scaffold density), but also note the false positive rate (which can differ from the missing branch rate when the supertrees are not fully resolved). In addition, we note the MRP and MRL scores; this allows us to consider whether using better heuristics (for the optimization problems) result in improved topological accuracy. Finally, we also examine running times.

#### Topological error rates

**Figure 1:**
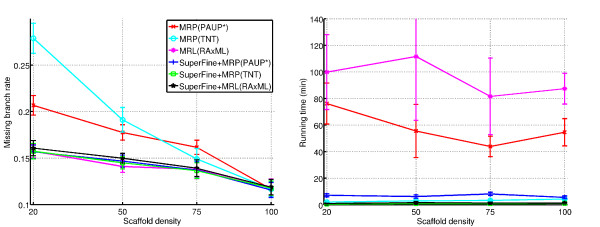
**Average missing branch rates and running times for 1000-taxon model conditions**. The average missing branch rates and running times (in minutes) for the supertree methods for the 1000-taxon model conditions as a function of scaffold density. The standard error is shown for the missing branch rates, and the standard deviation is shown for the running times. Averages are computed only on replicates where there is sufficient taxonomic overlap to perform an accurate supertree analysis. *n *= 10 for all scaffold densities except *n *= 7 for the 20% scaffold density, and *n *= 9 for 50% scaffold density.

We begin with a discussion of the missing branch rate for the estimated supertrees (Figure 1 and Table 2 for 1000-taxon datasets, and Table 3 for 500-taxon datasets). Note that the supertree methods had very close error rates when the scaffold tree contains all the taxa (i.e., the scaffold density is 100%), that all methods improved in accuracy as the scaffold density increases, and that the difference in error rates between methods decreased with the increase of scaffold density (trends already observed for SuperFine+MRP and other supertree methods in [17,28]). Since biological supertree datasets often do not contain scaffold trees containing all the taxa (and frequently contain only sparsely sampled scaffold trees), we focus our attention on performance on the sparse scaffold trees, with 20% or 50% scaffold densities.

**Table 2:** Missing branch rates for 1000-taxon model conditions

	Scaffold Density				
Method	20	50	75	100	Average

MRP(PAUP*)	20.7 (1.1)	17.7 (0.8)	16.2 (0.8)	11.7 (0.9)	16.2 (0.7)
MRP(TNT)	27.9 (1.6)	19.1 (1.3)	14.9 (1.1)	11.7 (0.8)	17.6 (1.1)

MRL(RAxML)	**15.7 (0.7)**	**14.1 (0.6)**	13.8 (1.0)	11.9 (0.8)	**13.7 (0.5)**

SCM	22.6 (0.7)	22.7 (0.7)	21.0 (0.7)	19.0 (0.6)	21.2 (0.4)

SuperFine+MRP(PAUP*)	**15.7 (0.7)**	14.7 (0.7)	13.7 (0.9)	**11.6 (0.8)**	**13.7 (0.5)**
SuperFine+MRP(TNT)	**15.7 (0.8)**	14.5 (0.7)	**13.6 (0.9)**	11.8 (0.8)	**13.7 (0.5)**
SuperFine+MRL(RAxML)	16.1 (0.8)	15.0 (0.5)	13.9 (0.9)	11.9 (0.8)	14.0 (0.5)

We show the average missing branch rates (reported as %) on the 1000-taxon datasets. Missing branch rate is calculated as total number of FN edges in the model tree divided by the total number of internal edges in the model tree. Each simulated dataset has 25 clade-based source trees and 1 scaffold tree. The scaffold density is the percentage of the full taxon set that is present in the scaffold tree. The standard error is shown in parenthesis. *n *= 7 for the 20% scaffold density, *n *= 9 for the 50% scaffold density, and *n *= 10 for the remaining scaffold densities. *n *= 36 for the average. The lowest missing branch rate for each scaffold density is shown in bold.

**Table 3:** Missing branch rates for 500-taxon model conditions

	Scaffold Density				
Method	20	50	75	100	Average

MRP(PAUP*)	22.1 (1.0)	18.8 (0.6)	14.7 (0.7)	**11.1 (0.4)**	16.4 (0.5)
MRP(TNT)	29.4 (1.7)	18.4 (0.9)	14.1 (0.6)	11.2 (0.4)	17.7 (0.8)

MRL(RAxML)	15.9 (0.6)	14.0 (0.5)	12.9 (0.5)	11.2 (0.4)	13.4 (0.3)

SCM	22.3 (0.6)	21.6 (0.5)	20.6 (0.6)	18.6 (0.6)	20.7 (0.3)

SuperFine+MRP(PAUP*)	15.2 (0.5)	14.0 (0.4)	12.5 (0.4)	**11.1 (0.4)**	13.1 (0.3)
SuperFine+MRP(TNT)	**15.0 (0.6)**	**13.9 (0.4)**	**12.4 (0.4)**	11.2 (0.4)	13.0 (0.3)
SuperFine+MRL(RAxML)	15.4 (0.5)	14.2 (0.4)	13.1 (0.4)	11.3 (0.4)	13.4 (0.3)

We present average missing branch rates (reported as %) on the 500-taxon datasets. Missing branch rate is calculated as total number of FN edges in the model tree divided by the total number of internal edges in the model tree. Each simulated dataset has 15 clade-based source trees and 1 scaffold tree. The scaffold density is the percentage of the full taxon set that is present in the scaffold tree. The standard error is shown in parenthesis. *n *= 24 for the 20% scaffold density, and *n *= 30 for the remaining scaffold densities. *n *= 114 for the average. The lowest missing branch rate for each scaffold density is shown in bold.

The next observation is that where there was any noticeable difference between supertree methods, MRP(TNT) and MRP(PAUP*) gave the highest missing branch rates, and that MRL(RAxML) and all versions of SuperFine gave the most accurate trees. Also, the difference between the error rates was greatest on the 20% scaffold density conditions, and decreased as the density of the scaffold tree increased, as expected.

An important observation is that although there were some statistically significant differences, the error rates of the various versions of SuperFine never differed by more than 1%. To some extent this is expected, since the majority of the SuperFine tree topology is produced by its first step, when it computes the SCM tree, and the second step (here performed using MRL or MRP) is limited to refining the SCM tree.

We now discuss the false positive rates; Table 4 gives these rates for 1000-taxon datasets and Table 5 gives rates for 500-taxon datasets. We note that the false positive and missing branch rates were nearly identical for the MRP and MRL trees, indicating that these trees were fully resolved. By contrast, the SuperFine trees were not always fully resolved, and hence had lower false positive rates than their missing branch rates.

**Table 4:** False positive rates for 1000-taxon model conditions

	Scaffold Density				
Method	20	50	75	100	Average

MRP(PAUP*)	20.7 (1.1)	17.7 (0.8)	16.1 (0.8)	11.7 (0.9)	16.2 (0.7)
MRP(TNT)	27.9 (1.6)	19.1 (1.3)	14.9 (1.1)	11.7 (0.8)	17.6 (1.1)

MRL(RAxML)	15.7 (0.7)	14.1 (0.6)	13.8 (1.0)	11.9 (0.8)	13.7 (0.5)

SCM	**5.9(0.5)**	**5.4(0.4)**	**4.9(0.6)**	**4.4(0.6)**	**5.1(0.3)**

SuperFine+MRP(PAUP*)	14.4 (0.6)	13.2 (0.6)	12.7 (0.8)	11.6 (0.8)	12.8 (0.4)
SuperFine+MRP(TNT)	14.4 (0.7)	13.0 (0.6)	12.6 (0.8)	11.8 (0.8)	12.8 (0.4)
SuperFine+MRL(RAxML)	14.8 (0.7)	13.5 (0.5)	12.9 (0.8)	11.9 (0.8)	13.1 (0.4)

We show average false positive rates (reported as %) on the 1000-taxon datasets. False positive rate is calculated as total number of FP edges in the estimated tree divided by the total number of internal edges in the internal tree. Each simulated dataset has 25 clade-based source trees and 1 scaffold tree. The scaffold density is the percentage of the full taxon set that is present in the scaffold tree. The standard error is shown in parenthesis. *n *= 7 for the 20% scaffold density, *n *= 9 for the 50% scaffold density, and *n *= 10 for the remaining scaffold densities. *n *= 36 for the average. The lowest false positive rate for each scaffold density is shown in bold.

**Table 5:** False positive rates for 500-taxon model conditions

	Scaffold Density				
Method	20	50	75	100	Average

MRP(PAUP*)	22.1 (1.0)	18.8 (0.6)	14.7 (0.7)	11.1 (0.4)	16.4 (0.5)
MRP(TNT)	29.4 (1.7)	18.4 (0.9)	14.1 (0.6)	11.2 (0.4)	17.7 (0.8)

MRL(RAxML)	15.9 (0.6)	14.0 (0.5)	12.9 (0.5)	11.2 (0.4)	13.4 (0.3)

SCM	**6.3(0.5)**	**5.9(0.4)**	**4.7(0.3)**	**4.0(0.3)**	**5.2(0.2)**

SuperFine+MRP(PAUP*)	13.9 (0.5)	12.6 (0.4)	11.5 (0.4)	11.1 (0.4)	12.2 (0.2)
SuperFine+MRP(TNT)	13.8 (0.6)	12.5 (0.4)	11.4 (0.4)	11.2 (0.4)	12.1 (0.2)
SuperFine+MRL(RAxML)	14.2 (0.5)	12.8 (0.4)	12.1 (0.4)	11.3 (0.4)	12.5 (0.2)

We present the average false positive rates (reported as %) on the 500-taxon datasets. False positive rate is calculated as total number of FP edges in the estimated tree divided by the total number of internal edges in the internal tree. Each simulated dataset has 15 clade-based source trees and 1 scaffold tree. The scaffold density is the percentage of the full taxon set that is present in the scaffold tree. The standard error is shown in parenthesis. *n *= 24 for the 20% scaffold density, and *n *= 30 for the remaining scaffold densities. *n *= 114 for the average. The lowest false positive rate for each scaffold density is shown in bold.

Finally, we discuss the SCM tree. We note that the missing branch rates (Tables 2 and 3) were quite high, and that the false positive rates (Tables 4 and 5) were quite low (although not equal to zero). The high false negative rate means that the SCM tree is not a good point estimate of the true tree, an observation also established in [24]. On the other hand, its low false positive rate means that most of its edges are likely to be true of the true tree, and makes it a good constraint tree (which is how it is used within SuperFine).

#### Which methods give good MRL and MRP scores?

With respect to MRL scores, not surprisingly, MRL(RAxML) gave the best MRL scores (Tables 6 and 7). The next best methods were SuperFine+MRP(TNT) and SuperFine+MRP(PAUP*). MRP(PAUP*) typically gave the least accurate MRL scores.

**Table 6:** MRL scores for 1000-taxon model conditions

	Scaffold Density				
Method	20	50	75	100	Average

MRP(PAUP*)	-16632 (1870)	-19844 (1988)	-21742 (2879)	-24325 (2896)	-20991 (3684)
MRP(TNT)	-16584 (1861)	-19764 (1983)	-21645 (2837)	-24332 (2896)	-20937 (3687)

MRL(RAxML)	**-16347 (1868)**	**-19700 (1996)**	**-21594 (2850)**	**-24288 (2898)**	**-20848 (3738)**

SuperFine+MRP(PAUP*)	-16368 (1869)	-19714 (1995)	-21625 (2844)	-24329 (2891)	-20876 (3742)
SuperFine+MRP(TNT)	-16366 (1870)	-19718 (1998)	-21630 (2845)	-24326 (2892)	-20878 (3742)
SuperFine+MRL(RAxML)	-16389 (1872)	-19749 (1996)	-21648 (2859)	-24336 (2894)	-20897 (3741)

True Tree	-16852 (1929)	-20246 (2024)	-22147 (2770)	-24820 (2783)	-21385 (3714)

We present the average MRL scores (ML scores under the symmetric two-state model with gamma-distributed rates across sites) with respect to the MRP matrix, given as log likelihood scores) for the 1000-taxon supertrees. Thus, numbers with smaller magnitude represent improvements. The scaffold density is the percentage of the full taxon set that is present in the scaffold tree. The standard deviation is shown in parenthesis. All scores are rounded to the nearest integer. The lowest MRL score (in magnitude) for each scaffold density is shown in bold.

**Table 7:** MRL scores for 500-taxon model conditions

	Scaffold Density				
Method	20	50	75	100	Average

MRP(PAUP*)	-7815 (2419)	-9089 (2377)	-10242 (2360)	-11425 (2349)	-9739 (2709)
MRP(TNT)	-7799 (2417)	-9039 (2373)	-10218 (2370)	-11426 (2349)	-9716 (2715)

MRL(RAxML)	**-7711 (2444)**	**-9013 (2376)**	**-10198 (2369)**	**-11408 (2347)**	**-9681 (2731)**

SuperFine+MRP(PAUP*)	-7721 (2449)	-9021 (2380)	-10209 (2374)	-11424 (2349)	-9692 (2736)
SuperFine+MRP(TNT)	-7722 (2449)	-9022 (2380)	-10209 (2373)	-11426 (2351)	-9693 (2736)
SuperFine+MRL(RAxML)	-7731 (2449)	-9035 (2390)	-10221 (2377)	-11433 (2351)	-9704 (2740)

True Tree	-7901 (2454)	-9216 (2377)	-10390 (2376)	-11607 (2346)	-9877 (2736)

We present the average MRL scores (ML scores with respect to the MRP matrix, given as log likelihoods) for the 500-taxon supertrees. Thus, numbers with smaller magnitude represent improvements. The scaffold density is the percentage of the full taxon set that is present in the scaffold tree. The standard deviation is shown in parenthesis. All scores are rounded to the nearest integer. The lowest MRL score (in magnitude) for each scaffold density is shown in bold.

With respect to MRP scores (Tables 8 and 9), we find that SuperFine+MRP(TNT) and SuperFine+MRP(PAUP*) had nearly identical performance and the best scores of all methods for scaffold densities of 20% and 50%. For denser scaffolds, MRP(TNT), SuperFine+MRP(TNT), and SuperFine+MRP(PAUP*) had the best scores. At 100% scaffold density, MRP(PAUP*) also produced the best scores. Thus, although MRP(PAUP*) and MRP(TNT) directly try to optimize MRP scores, SuperFine+MRP(PAUP*) and SuperFine+MRP(TNT) gave better MRP scores, and hence were more effective heuristics for MRP, especially for sparse scaffolds.

**Table 8:** MRP scores for 1000-taxon model conditions

	Scaffold Density				
Method	20	50	75	100	Average

MRP(PAUP*)	2653 (276)	2981 (290)	3141.30 (428)	**3390 (423)**	3075 (449)
MRP(TNT)	2625 (268)	2962 (292)	**3120 (421)**	**3390 (423)**	3059 (452)

MRL(RAxML)	2618 (275)	2969 (293)	3143 (417)	3424 (429)	3075 (463)

SuperFine+MRP(PAUP*)	**2612 (275)**	**2960 (294)**	**3120 (421)**	**3390 (423)**	**3056 (456)**
SuperFine+MRP(TNT)	2614 (276)	**2960 (294)**	**3120 (421)**	**3390 (423)**	**3056 (456)**
SuperFine+MRL(RAxML)	2628 (277)	2980 (294)	3141 (421)	3407 (425)	3075 (457)

True Tree	2788 (294)	3144 (307)	3308 (411)	3578 (405)	3241 (456)

We present the average MRP scores (MP scores with respect to the MRP matrix) for the 1000-taxon supertrees. The scaffold density is the percentage of the full taxon set that is present in the scaffold tree. The standard deviation is shown in parenthesis. All scores are rounded to the nearest integer. The lowest MRP score for each scaffold density is shown in bold.

**Table 9:** MRP scores for 500-taxon model conditions

	Scaffold Density				
Method	20	50	75	100	Average

MRP(PAUP*)	1283 (330)	1434 (334)	1563 (332)	**1694 (336)**	1504 (364)
MRP(TNT)	1273 (331)	1422 (334)	**1556 (335)**	**1694 (336)**	1497 (367)

MRL(RAxML)	1276 (338)	1431 (336)	1570 (336)	1707 (339)	1508 (372)

SuperFine+MRP(PAUP*)	**1268 (334)**	1422 (334)	1557 (335)	**1694 (336)**	**1496 (369)**
SuperFine+MRP(TNT)	**1268 (334)**	**1421 (334)**	**1556 (335)**	**1694 (337)**	**1496 (369)**
SuperFine+MRL(RAxML)	1277 (337)	1431 (341)	1568 (340)	1704 (340)	1507 (373)

True Tree	1347 (341)	1502 (337)	1634 (340)	1773 (339)	1575 (372)

We show the average MRP scores (MP scores with respect to the MRP matrix) for the 500-taxon supertrees. The scaffold density is the percentage of the full taxon set that is present in the scaffold tree. The standard deviation is shown in parenthesis. All scores are rounded to the nearest integer. The lowest MRP score for each scaffold density is shown in bold.

These results together suggest that the best MRP heuristics are SuperFine+MRP(PAUP*) and SuperFine+MRP(TNT) (followed by MRP(TNT)), while the method that generally produces the best MRL scores is MRL(RAxML), followed closely by SuperFine+MRP(PAUP*) and SuperFine+MRP(TNT).

#### Running Time

In many ways, the different variants of SuperFine are extremely close, producing trees of almost identical topological accuracy (among the most accurate across all scaffold densities), and producing good heuristics for MRP and MRL. However, how do they perform in terms of running time? We focus here on the results for the 1000-taxon datasets, shown in Figure 1 and Table 10 (see Table 11 for 500-taxon datasets). All versions of the SuperFine methods were fast, finishing in all the simulated datasets in just a few minutes (on average, about eight (8) minutes on the 1000-taxon datasets). MRP(TNT) was also fast (finishing in under 5 minutes on all these datasets), but the remaining methods were much slower: MRL(RAxML) often took more than 1.5 hours and MRP(PAUP*) took close to an hour on the 1000-taxon datasets.

**Table 10:** Running times for 1000-taxon model conditions

	Scaffold Density				
Method	20	50	75	100	Average

MRP(PAUP*)	76.14 (15.45)	55.53 (19.99)	43.87 (7.72)	54.56 (10.24)	56.03 (17.67)
MRP(TNT)	2.01 (0.64)	2.95 (1.31)	3.27 (0.59)	4.33 (0.75)	3.24 (1.19)

MRL(RAxML)	99.86 (28.15)	111.57 (48.06)	81.57 (28.87)	87.33 (11.59)	94.22 (33.76)

SuperFine+MRP(PAUP*)	7.14 (1.10)	6.16 (1.28)	8.13 (1.27)	5.56 (1.09)	6.73 (1.57)
SuperFine+MRP(TNT)	**0.54 (0.11)**	**0.80 (0.09)**	**0.84 (0.16)**	**1.07 (0.17)**	**0.83 (0.23)**
SuperFine+MRL(RAxML)	1.00 (0.14)	1.56 (0.94)	1.32 (0.71)	1.27 (0.23)	1.30 (0.64)

We show the average running times, in minutes, to calculate the 1000-taxon supertrees estimated from 25 clade-based source trees and 1 scaffold tree. The standard deviation is shown in parenthesis. The lowest running time for each scaffold factor is shown in bold.

**Table 11:** Running times for 500-taxon model conditions

	Scaffold Density				
Method	20	50	75	100	Average

MRP(PAUP*)	8.98 (1.66)	8.96 (1.43)	9.58 (2.28)	8.12 (1.63)	8.91 (1.86)
MRP(TNT)	0.32 (0.15)	0.42 (0.12)	0.45 (0.11)	0.53 (0.10)	0.43 (0.14)

MRL(RAxML)	18.99 (6.88)	19.24 (4.72)	20.35 (5.96)	18.35 (4.84)	19.24 (5.65)

SuperFine+MRP(PAUP*)	4.75 (1.46)	4.30 (1.23)	3.24 (1.89)	5.87 (1.53)	4.53 (1.82)
SuperFine+MRP(TNT)	**0.22 (0.09)**	**0.27 (0.08)**	**0.31 (0.07)**	**0.41 (0.23)**	**0.30 (0.15)**
SuperFine+MRL(RAxML)	0.40 (0.13)	0.49 (0.12)	0.58 (0.13)	0.55 (0.31)	0.51 (0.20)

We give the average running times, in minutes, to calculate the 500-taxon supertrees estimated from 15 clade-based source trees and 1 scaffold tree. The standard deviation is shown in parenthesis. The lowest running time for each scaffold factor is shown in bold.

**Figure 2:**
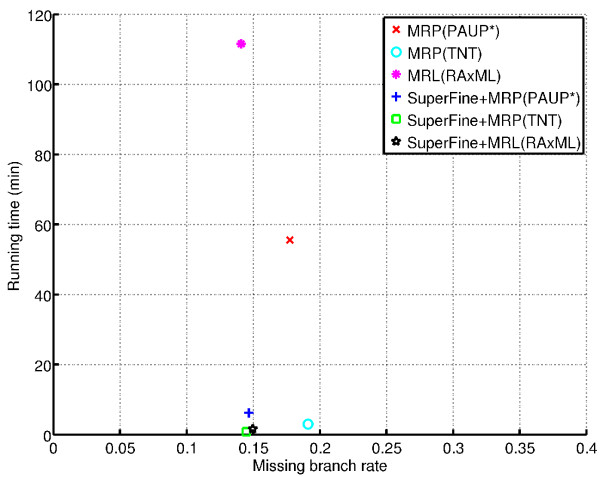
**Scatterplot of average missing branch rates versus running times for 1000-taxon 50% scaffold density model conditions**. The average missing branch rate versus running times (in minutes) for the supertree methods for 9 replicates of the 1000-taxon 50% scaffold density model conditions.

The tradeoffs between the different methods can be seen more clearly on the sparse scaffold conditions. Figure 2 shows that MRL(RAxML) was slow but very accurate, MRP(TNT) was very fast but inaccurate, MRP(PAUP*) fell in between both of these methods, and all SuperFine methods were both accurate and fast.

### Correlation of MRL, MRP, and Sum-FN with tree error

We also consider the question of how well the MRL, MRP, and Sum-FN scores correlate with tree error (as measured by FN rate). In other words, is it helpful to find a supertree that optimizes MRL, MRP, or Sum-FN?

Some trends are immediately obvious from the correlation analysis (Tables 12 and 13). First, all three scores were statistically correlated with tree error according to Spearman's rank correlation test and after Bonferroni correction for multiple hypothesis testing (p-values not shown). Second, Sum-FN and MRP scores had roughly the same correlation coefficient across the scaffold densities. Focusing on low scaffold densities, MRL had a much larger correlation coefficient than MRP and Sum-FN. In general, MRL and tree error were more strongly correlated at all scaffold densities, except at 100% scaffold densities, where all scores correlated strongly with tree error. We also note that all pairwise correlations became stronger as the scaffold density increased.

**Table 12:** Correlation analyses for 1000-taxon model conditions

	Scaffold Density			
Statistic	20	50	75	100

MRP Score	0.770	0.908	0.968	0.991
MRL Score	**0.871**	**0.935**	**0.976**	0.988
Sum-FN	0.762	0.907	0.966	**0.992**

We show the average Spearman's rank correlation coefficient between different statistics and the FN error rates of trees generated around each of the six estimated supertrees for the 1000-taxon model conditions. For each of the estimated six supertrees, 100 trees were generated using a p-ECR move, for a total of 606 trees (600 *p*-ECR trees plus 6 supertrees) per replicate. MRP score is the MP score of the estimated tree with respect to the MRP matrix. MRL score is the negative log-likelihood score of the estimated tree with respect to the MRP matrix. Sum-FN is the sum of the bipartitions in the source trees not present in the estimated tree divided by the total number bipartitions in the source trees. Coefficients with larger magnitude represent stronger correlation between the test statistic and FN error rates. The largest correlation coefficient for each scaffold density is shown in bold.

**Table 13:** Correlation analyses for 500-taxon model conditions

	Scaffold Density			
Statistic	20	50	75	100

MRP Score	0.690	0.879	0.947	0.984
MRL Score	**0.825**	**0.913**	**0.957**	0.980
Sum-FN	0.689	0.879	0.948	**0.985**

We show the average Spearman's rank correlation coefficient between different statistics and the FN error rates of trees generated around each of the six estimated supertrees for the 500-taxon model conditions. For each of the six estimated supertrees, 100 trees were generated using a p-ECR move, for a total of 606 trees (600 *p*-ECR trees plus 6 supertrees) per replicate. MRP score is the MP score of the estimated tree with respect to the MRP matrix. MRL score is the negative log-likelihood score of the estimated tree with respect to the MRP matrix. Sum-FN is the sum of the bipartitions in the source trees not present in the estimated tree divided by the total number bipartitions in the source trees. Coefficients with larger magnitude represent stronger correlation between the test statistic and FN error rates. The largest correlation coefficient for each scaffold density is shown in bold.

This preliminary study showing a relatively strong correlation between MRL scores and tree error suggests that methods for optimizing MRL scores may have some inherent value, especially for the low scaffold density conditions where the correlation between MRP scores and tree error is much lower.

### Biological datasets

Because we do not have a reliable "true species tree" for the biological datasets, we compare estimated supertrees in terms of Sum-FN, MRP, and MRL scores. While we have shown the correlation of these scores to topological error for the simulated datasets, the extent of correlation for each empirical dataset is not known. Nevertheless, these scores, when considered together, enable a framework, albeit an imperfect one, for evaluating estimated supertrees. Table 1 shows the reference for each biological dataset and various empirical statistics (number of source trees, number of taxa, the scaffold density, degree of resolution for the SCM tree). We show Sum-FN scores in Table 14, MRP scores in Table 15, MRL scores in Table 16, and running time in Table 17.

**Table 14:** Sum-FN rates for the biological supertrees

	Biological Dataset				
Method	Placental	Seabirds	Marsupials	THPL	CPL

MRP(PAUP*)	**35.8**	16.0	26.0	25.1	33.3
MRP(TNT)	**35.8**	13.8	26.0	21.3	33.2

MRL(RAxML)	36.3	21.0	26.2	16.2	35.1

SuperFine+MRP(PAUP*)	**35.8**	13.8	26.0	16.7	**33.1**
SuperFine+MRP(TNT)	**35.8**	**12.7**	**25.8**	**15.8**	33.4
SuperFine+MRL(RAxML)	36.3	16.0	26.8	18.9	34.2

The lowest Sum-FN for each dataset is shown in bold.

**Table 15:** The MRP scores (MP scores with respect to the MRP matrix) for the biological supertrees

	Biological Dataset				
Method	Placental	Seabirds	Marsupials	THPL	CPL

MRP(PAUP*)	**9486**	217	**2273**	974	5488
MRP(TNT)	**9486**	**213**	**2273**	931	5477

MRL(RAxML)	9508	230	2286	890	5738

SuperFine+MRP(PAUP*)	**9486**	214	**2273**	902	5481
SuperFine+MRP(TNT)	**9486**	**213**	**2273**	**881**	**5475**
SuperFine+MRL(RAxML)	9508	220	2295	911	5671

The lowest MRP score for each dataset is shown in bold.

**Table 16:** The MRL scores (ML scores with respect to the MRP matrix, given as log likelihoods) for the biological supertrees

	Biological Dataset				
Method	Placental	Seabirds	Marsupials	THPL	CPL

MRP(PAUP*)	-41544	-1137	-10977	-5182	-41003
MRP(TNT)	-41544	-1124	-10974	-5043	-41053

MRL(RAxML)	**-41483**	**-1113**	**-10959**	**-4749**	**-40080**

SuperFine+MRP(PAUP*)	-41543	-1124	-10974	-4845	-40890
SuperFine+MRP(TNT)	-41546	-1120	-10968	-4800	-40923
SuperFine+MRL(RAxML)	**-41483**	-1128	-10980	-4799	-40533

Numbers with smaller magnitude represent improvements. All scores are rounded to the nearest integer. The lowest MRL score (in magnitude) for each dataset is shown in bold.

**Table 17:** Running times, in minutes, for the biological supertrees

	Biological Dataset				
Method	Placental	Seabirds	Marsupials	THPL	CPL

MRP(PAUP*)	3.57	0.22	3.87	31.97	675.00
MRP(TNT)	**0.13**	**0.02**	**0.18**	0.38	29.82

MRL(RAxML)	7.47	0.45	7.20	25.37	461.82

SuperFine+MRP (PAUP *)	4.00	0.20	2.60	0.72	21.97
SuperFine+MRP(TNT)	1.30	0.07	0.67	**0.15**	**3.48**
SuperFine+MRL(RAxML)	9.23	0.05	5.00	0.47	29.02

The lowest running time for each dataset is shown in bold.

#### Placental dataset

All methods resulted in identical Sum-FN scores on this dataset. Unsurprisingly, MRP and SuperFine+MRP methods resulted in the best MRP scores, and MRL(RAxML) and SuperFine+MRL(RAxML) in the best MRL scores. The fastest method was MRP(TNT) (less than a minute) followed closely by SuperFine+MRP(TNT) (less than two minutes), and only the methods that use RAxML took more than five minutes. Due to the large number of source trees, many of which had incompatible edges, the SCM tree was almost completely unresolved. For this dataset, therefore, the SuperFine trees were almost identical to trees obtained using their base supertree methods.

#### Seabirds dataset

SuperFine+MRP and MRP(TNT) had the best MRP scores and the best Sum-FN scores. MRL(RAxML) had the best MRL score, followed by SuperFine+MRP(TNT). All methods completed in under a minute for this dataset.

#### Marsupials dataset

On this dataset, all methods had close Sum-FN scores. MRP and SuperFine+MRP again had the best MRP scores, and MRL(RAxML) had the best MRL score followed by SuperFine+MRP(TNT). Both MRP(TNT) and SuperFine+MRP(TNT) completed in under a minute, while MRL(RAxML) (the slowest method) completed in under eight minutes.

#### THPL dataset

For this dataset, SuperFine methods and MRL(RAxML) resulted in much better Sum-FN scores compared to the other methods. SuperFine+MRP(TNT) resulted in the best MRP score, followed closely by MRL(RAxML). An interesting observation is that MRP methods had, by far, the worse MRP scores. MRL(RAxML) once again resulted in the best MRL score, followed by SuperFine+MRL(RAxML) and SuperFine+MRP(TNT). All SuperFine methods and MRP(TNT) completed in under a minute, but MRL(RAxML) and MRP(PAUP*) were much slower (25 and 32 minutes, respectively).

#### CPL dataset

All methods resulted in similar Sum-FN scores. The best MRP scores were obtained by SuperFine+MRP(TNT) and MRP(TNT). The best MRL score was obtained by MRL(RAxML), followed by SuperFine+MRL(RAxML). This dataset is the largest dataset we examined, with 2,228 taxa and 39 source trees, and the six supertree methods differed substantially in terms of their running times. MRL(RAxML) and MRP(PAUP*) were the slowest, finishing in 461 minutes (i.e., more than 7.5 hours) and 675 minutes (i.e., more than 11 hours), respectively. MRP(TNT) and SuperFine+MRL(RAxML) were the next slowest, finishing in 30 minutes and 29 minutes, respectively. By comparison, SuperFine+MRP(TNT) completed in less than 4 minutes. Thus, only SuperFine+MRP(TNT) was fast on this dataset.

#### Summary

Several key observations are noted. First, SuperFine+TNT gave the best results for Sum-FN, but other than on the seabirds and THPL datasets, all methods produced trees with similar Sum-FN scores. MRL(RAxML) typically resulted in the best MRL scores, and SuperFine+MRP(TNT) often produced trees with the second best MRL scores. SuperFine+MRP(TNT) also resulted in the best MRP scores for all datasets. SuperFine+MRP(TNT) was among the fastest methods, and on the largest dataset it was substantially faster than any other method. Thus, although we cannot evaluate the topological accuracy of any of these estimated supertrees, SuperFine+MRP(TNT) had a good overall performance for all criteria we evaluate (MRP, MRL, Sum-FN, and running time).

## Discussion and Conclusion

Supertree estimation methods need to be both highly accurate and also reasonably fast, as otherwise they will not be useful in estimating large phylogenies. Our discussion thus addresses both running time and topological accuracy.

The results for the simulated datasets show clearly that all the methods produce trees with about the same accuracy on datasets with very dense scaffolds, but differ substantially in terms of accuracy on the datasets with sparser scaffolds. Since sparser scaffolds are common for biological supertree inputs, the differences in accuracy on sparse scaffolds is important.

In general we found that all the SuperFine variants we studied (whether using MRL or MRP to refine polytomies in the SCM tree) produced very accurate trees, and that differences between them were largely in terms of running time, or with respect to MRL, MRP, or Sum-FN score. With respect to running time, SuperFine+MRP(TNT) was the fastest of all the methods we studied, finishing in at most 4 minutes on all the datasets (including the largest one with 2228 taxa and 39 source trees). Furthermore, SuperFine+MRP(TNT) produced very good MRP and MRL scores, outperforming TNT and PAUP* with respect to MRP score optimization. On the biological datasets, we also observed similar results, including that SuperFine+MRP(TNT) generally produced very good Sum-FN scores. Thus, although SuperFine+MRP(TNT) was not designed to be a heuristic for any of these criteria, it has excellent performance across the board.

It is worth discussing in greater depth the results we showed for Sum-FN scores. Our study shows that neither MRP nor Sum-FN scores have the best correlation with tree error, except when the scaffold factor is very dense. This result suggests that optimizing MRP or Sum-FN may not be the best strategy (except with dense scaffolds), and that evaluating supertree methods with respect to Sum-FN may not be the best way of distinguishing methods (except for dense scaffold datasets, perhaps). These observations were made earlier in [24], but are worth repeating here, because of the increased interest in an approach to supertree estimation proposed by Steel and Rodrigo [15], called "maximum likelihood supertrees". This method is based upon an exponential error model, and can be based upon different ways of measuring distances between trees and weights on the input trees. However, in the simplest case, where the weights on trees are all the same and the distance between trees is the RF distance, finding the ML supertree is identical to optimizing Sum-RF (minimizing the total topological distance, using Robinson-Foulds scores, to the input trees), a criterion that is almost identical to Sum-FN. Indeed, when the input estimated trees are binary, these criteria are exactly the same. Since our simulation study estimated supertrees from binary source trees, our correlation analysis also shows that optimizing Sum-RF is not likely to be the best strategy, except for dense scaffold datasets, and thus suggests that the use of RF distance metric within the ML supertree approach proposed by Steel and Rodrigo may not be appropriate. We note here a potential shortcoming of the ML supertree approach in general: it seems likely that the probability of a particular estimated tree will not depend only on the topological distance it has to the true tree, but rather also on the parameters of the true tree (especially the branch lengths), since very short branches are more likely to fail to be recovered in a phylogenetic estimation than longer branches.

A fundamental observation in this study is that searching for supertrees that optimize the maximum likelihood score under the S2+CAT model improved tree accuracy, a trend that we found quite surprising. The MRP matrix is a collection of partial binary characters defined by the input source trees. When these trees are compatible, the MRP matrix will exhibit no homoplasy at all, a condition under which the MRP solution will yield the true tree. Therefore, when there is no homoplasy, the ML solution under a no-common-mechanism model [33] (in which every combination of edge and site has its own rate parameter) will also produce the true tree, since then ML and MP produce the same trees. However, standard ML models (including the model used in this study) assume *i.i.d*. rates across sites, which does not yield the same result. Thus, we do not have a theoretical explanation for why optimizing likelihood under S2+CAT should lead to good supertrees. All we can say is that the data suggest that there *may *be some value (even if only approximate, and perhaps only under some conditions, not yet understood) in using maximum likelihood under this model as an optimization criterion for estimating supertrees. Future work should investigate whether optimizing the MRL score continues to return good solutions when the source trees are estimated from sequences that evolve under more realistic conditions, including indels, heterotachy, and non-stationarity.

As has been noted in [34], supertree analyses are not always able to completely identify the true tree, because the conditions required for such identification include correct source trees and overlap properties that may not be true of any given set of source trees. However, alternatives - such as combined analyses, in which a phylogeny estimation method is applied to a concatenation of the gene sequence alignments - also have only limited guarantees. From a practical standpoint, the evidence suggests that while combined analyses can yield more accurate trees [20,24] than supertree methods, there are conditions in which combined analysis methods cannot be used (e.g., heterogeneous data, including morphology, gene orders, or different types of molecular data), or are simply too computationally intensive. In these cases, improved supertree methods can be important tools in the phylogenetics toolkit.

In summary, this study introduces a new set of supertree methods based upon combining the divide-and-conquer strategy within SuperFine with fast supertree methods. In particular, the combination of SuperFine with TNT is extremely fast and produces very accurate supertrees, even on the largest datasets we studied. Earlier work [24] showed that SuperFine (based upon MRP, and using PAUP*) came very close to the accuracy of combined analysis based upon maximum likelihood. Future work should investigate statistical approaches to supertree estimation (along the lines of maximum likelihood supertrees, but taking branch lengths or support into account). The combination of SuperFine with such statistically-based supertree methods might close the gap between combined analysis and supertree methods.

## Competing interests

The authors declare that they have no competing interests.

## Authors' contributions

TW designed the study; NN and SM developed the software; NN performed the study; TW, NN, and SM analyzed the data and wrote the paper. All authors read and approved the final manuscript.
